# Psychometric Properties of the Greek Version of the Pediatric Assessment Scale for Severe Feeding Problems (PASS-FP)

**DOI:** 10.3390/medsci13030133

**Published:** 2025-08-14

**Authors:** Andri Papaleontiou, Louiza Voniati, Vassiliki Siafaka, Alexandros Gryparis, Rafaella Georgiou, Dionysios Tafiadis

**Affiliations:** 1 Department of Speech & Language Therapy, School of Health Sciences, University of Ioannina, 45500 Ioannina, Greece; siafaka@uoi.gr (V.S.); alexandros@uoi.gr (A.G.); r.georgiou@uoi.gr (R.G.); tafiadis@uoi.gr (D.T.); 2 Department of Health Sciences, Speech and Language Therapy, European University, 2404 Nicosia, Cyprus; l.voniati@euc.ac.cy

**Keywords:** childhood feeding difficulties, parent report instrument, pediatric assessment scale for severe feeding problems (PASS-FP), psychometric properties

## Abstract

**Background/Objective:** Pediatric feeding problems are becoming more widespread. They represent a synthesis of social, behavioral, and biological issues. Inevitably, the multifaceted nature of these problems has to be evaluated through one multidimensional tool. The Pediatric Assessment Scale for Severe Feeding Problems is designed to assess these complex issues and represents the first attempt to associate these factors into a single multidimensional measure. The aim of this study is to translate and culturally adapt the Pediatric Assessment Scale for Severe Feeding Problems into Greek and to assess its psychometric properties for use among a Greek Cypriot pediatric sample. **Methods:** This study involved 142 parents of children with symptoms of feeding problems. It included parents of children who were oral-fed (*n* = 65), partially oral-fed with supplementation (*n* = 62), and tube-fed (*n* = 15). The sample of parents was recruited from mainstream and special education schools in Cyprus and were asked to complete a Greek-translated version of the PASS-FP. **Results:** The PASS-FP-Gr demonstrated excellent psychometric properties. Internal consistency was good, and test–retest reliability showed a perfect Spearman’s rank correlation with high significance. The tool exhibited strong discriminatory ability, with statistically significant differences in median scores across the three feeding groups. **Conclusions:** The PASS-FP appears sensitive to the Greek Cypriot population and presents satisfactory psychometric features. It demonstrated excellent discriminatory ability, as evidenced by the participants’ consistent response patterns.

## 1. Introduction

Feeding difficulties arise from a complex interplay of social, behavioral, and biological factors that interfere with a child’s ability to feed successfully and meet nutritional needs. These challenges are more often observed in the pediatric population, with prevalence estimates ranging from 25% to 35% in developmentally typical children [1,2,3]. The prevalence increases significantly—up to 40–70%—in children with developmental disabilities and chronic medical conditions [4]. The underlying causes of feeding difficulties vary, including neurodevelopmental disorders, conditions of appetite regulation, metabolic diseases, sensory defects, conditioned dysphagia, and anatomical abnormalities [5]. While these issues may arise from disease, behavioral factors also significantly contribute to their development [6,7]. Recent findings suggest that concomitant conditions—such as gastrointestinal disorders, neurological impairments, metabolic diseases, and sensory processing issues—play a significant role in exacerbating feeding challenges, especially in pediatric populations. Children with conditions like autism spectrum disorder (ASD) and cerebral palsy often experience difficulties with oral intake, texture aversion, or swallowing. Thus, the interplay between associated conditions and feeding challenges can complicate the feeding processes, leading to adverse outcomes such as malnutrition, growth delays, and increased parent/caregiver stress [8,9,10,11]. Furthermore, new research has also highlighted a spectrum of difficulties in terms of their kind and intensity, which could be used to characterize feeding and swallowing challenges for young children [12].

Due to risks such as aspiration, the degree of feeding difficulty ranges from “picky eating” toddlers to tube-fed children for whom oral feeding is intricate [13]. Also, many children with cerebral palsy and children with feeding challenges may have significant sensory or oral motor difficulties, or they may not have any trouble at all [14,15]. It is well known that dealing with feeding difficulties in children requires intervention and the integrated collaboration of several specialties to ensure effective therapeutic intervention [16,17]. However, despite this multidisciplinary–interdisciplinary collaboration, standardized and comprehensive assessment tools remain essential to address individualized needs in terms of oral and motor development, sensory processing, and nutritional adequacy, as well as the diverse behaviors of children and parents during mealtime [18].

Several contemporary theoretical models provide frameworks for understanding feeding difficulties in children. One widely recognized approach is the biopsychosocial model, which considers how medical conditions, behavioral patterns, and a child’s environment interact to influence feeding [19]. Behavioral models, such as those grounded in applied behavior analysis (ABA), focus on learned feeding patterns and reinforcement strategies [20]. The neurodevelopmental perspective highlights the role of sensory processing and motor coordination in feeding, especially relevant for children with developmental disabilities [21]. Another well-established framework is Ellyn Satter’s Division of Responsibility model which promotes the importance of clearly defined roles between the parent/caregiver and child during mealtimes, promoting autonomy and reducing stress [22].

Gastrostomy Tube (GT) feeding ensures the body receives vital nutrition and fluids [23]. It results from oral feeding resistance in children or adverse feeding experiences (such as choking, gagging, or vomiting) brought on by biological, social/behavioral, or medical causes. GT feeding is vital for children whose feeding issues are severe enough to adversely affect their growth, nutritional status, and failure to thrive [24]. As the ultimate objective of treatment is to restore or reintroduce oral eating gradually, this may have significant immediate benefits. Although GT feedings ensure sufficient nutrition and hydration, they may prevent the improvement of oral feeding by decreasing the child’s hunger-driven motivation to eat, thus reducing oral stimulation [25,26].

At the same time, as reported by Twachtman-Reilly, Amaral, and Zebrowski (2008), children diagnosed with autism spectrum disorder (ASD) may present particular difficulties in feeding [27]. Referring to the causes that may affect feeding in children with ASD, they emphasize that feeding difficulties in these children may be based on sensory processing, where the child “filters” and chooses from the multitude of information what to eat, choosing from the smell, the quantity and quality, the color of the food [27,28]. In addition, the current evidence base on feeding difficulties in children with ASD primarily emphasizes behavioral factors. Children with ASD frequently exhibit specific food preferences and rigid routines related to food preparation and mealtime behaviors. Studies indicate that positive feeding interactions are more likely when the environment is structured to meet their unique needs. Factors such as reduced ambient noise, familiar food types, consistent mealtime rules, and minimized sensory stimuli appear to contribute to more comfortable and successful feeding experiences in this population [27,29].

Importantly, the identification of feeding difficulties and the implementation of therapeutic interventions depend significantly on the assessment tools employed. Assessment tools play a critical role in clinical decisions and offer insights into parent–child interactions during feeding process. Such interactions are critical, as children with ASD often rely on their parents for both nourishment and support in navigating mealtime routines [30]. To address these challenges effectively, it is essential to implement a comprehensive therapeutic strategy that integrates dietary, medical, nutritional, and psychological interventions [18].

Parent/caregiver report questionnaires serve as vital tools in the assessment of pediatric feeding disorders, providing valuable insights into feeding behaviors, mealtime dynamics, and the parent–child relationship [2,31]. They can offer a more comprehensive understanding of the child’s feeding challenges by capturing contextual factors that may not be evident through direct observation alone [32]. Such questionnaires will allow the clinician to obtain comprehensive information efficiently and enable the early identification and intervention of feeding disorders [33]. Parental/caregiver reports contribute valuable insights into both typical and atypical mealtime performance and abilities, supporting the development of more tailored and effective intervention strategies [34].

While there are several parent report questionnaires for assessing feeding difficulties in children, such as the Behavioral Pediatrics Feeding Assessment Scale (BPFAS) and the Montreal Children’s Hospital Feeding Scale (MCHFS), the Pediatric Assessment Scale for Severe Feeding Problems (PASS-FP) is a parent report reliable assessment scale for severe feeding disorders in children. It investigates the medical, nutritional, and psychological aspects of feeding, together with the specific feeding methods utilized [18].

The present study aimed to translate the PASS-FP into Greek and examine its psychometric properties within the Greek Cypriot pediatric population, particularly among parents of children with various feeding deficits. Currently, there are no valid Greek questionnaires for assessing severe feeding difficulties in children, including those dependent on GT feeding and those with moderate feeding or swallowing impairments. This highlights a significant gap in the literature and underscores the relevance of the current study. Moreover, the PASS-FP is a valuable tool for evaluating the progress of oral feeding skills in children with neurological disorders who rely on tube feeding, as was the initial intent of the PASS-FP questionnaire.

A recent study by Yazici-Gulay et al. (2021) evaluated the Turkish version of the PASS-FP, confirming its reliability and validity [3]. The questionnaire includes 15 items across Forms A and B [3]. Form A was distributed to all participating parents to evaluate children’s eating behaviors and sensory responses, including their perception of eating as an enjoyable activity and reactions to various tastes and textures. Form B was administered exclusively to parents of children who were partially or fully orally fed. This form focused on sensory responses, such as the child’s positive reaction to brushing or rubbing their teeth and gums with a cloth; behavioral responses, including their acceptance of a spoon; and qualitative aspects of mealtime, such as the parent’s enjoyment of feeding their child. These forms thus provided a systematic method of evaluating feeding experiences in children and established the extent of feeding difficulties [3].

## 2. Materials and Methods

### 2.1. Participants

The participants in this study were parents of 142 young children with various neurological and medical diagnoses. Eligibility criteria for participation included (A) a confirmed diagnosis of a feeding disorder in their child, (B) willingness to provide informed consent, (C) sufficient ability to understand, speak, and read the Greek language, and (D) having a child between the ages of 6 months and 16 years.

These children were classified into three distinct feeding subgroups: orally fed, partially orally fed with supplementation, and tube-fed. The sample included children with neurological disorders (such as cerebral palsy and gastric reflux disease), prematurity (often associated with cardio-respiratory problems), gastrointestinal issues (such as reflux disease), and other medical conditions. A specific inclusion criterion was that the GT-fed participants had to be at least four months old, and the duration of GT feeding had to be at least four months [18]. A purposive sampling method was used to ensure the inclusion of children with a wide range of feeding difficulties. Participants were recruited from various public mainstream and special schools and private speech–language therapy clinics in Cyprus.

The results of this study revealed significant variations across the groups that included individuals undergoing partial tube feeding, partial oral feeding, and complete oral feeding [18].

### 2.2. Procedure

A copyright license was obtained by William Christ, author and creator of the PASS-FP, to translate and validate the questionnaire [18]. Ethical approval was granted by Cyprus (EEBK/E/2019/95) and Greece’s Bioethics Committees (with Approval Number 27829).

**Figure 1:**
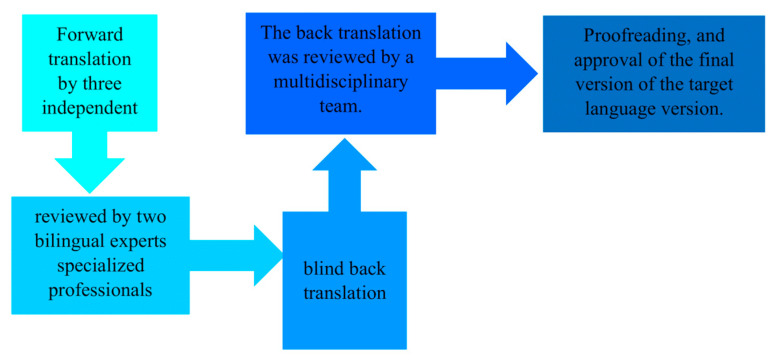
Translation process.

The original PASS-FP was first translated into Greek and then back-translated into English by an independent third party to ensure the original meanings of the items were accurately preserved. The steps are illustrated in Figure 1, as defined by the WHO, 2020 [35]. Participants were recruited from various public mainstream and special schools and from private speech–language therapy clinics in Cyprus. The questionnaire was completed by parents/caregivers using the paper–pencil method in a quiet setting, and the average completion time was approximately 10 min. To examine test–retest reliability, all parents were requested to complete the questionnaire a second time in ten days. A pilot study was also conducted to assess the adequacy of the Greek version of the questions based on the time of administration, completion rates, and score distributions [36].

### 2.3. Instrument

The Pediatric Assessment Scale for Severe Feeding Problems (PASS-FP), developed by Crist and colleagues in 2004, consists of 15 items to assess nutritional, oral sensory, oral motor, behavioral feeding, and quality-of-life challenges. It includes two main forms: Form A and Form B. Form A is completed for all children and covers general feeding and nutritional concerns, while Form B is completed only by parents of children who are partially or fully orally fed and includes more specific behavioral, sensory and quality-of-life issues. Items in Form A include the first nine questions, while Form B contains six items. The first three questions are scored as follows: Question 1 is scored from 0 to 5 based on the child’s feeding description, Question 2 from 0 to 4 depending on tube versus oral feeding, and Question 3 from 0 to 9 based on swallowing ability. The remaining 12 items use a consistent 5-point Likert scale ranging from 0 (never/does not apply) to 4 (always), with higher scores indicating better functional feeding performance. Thus, the scores range from 0 to 66, with lower scores reflecting more severe feeding difficulties.

In this study, three derived scores were used for analysis: (1) Total_score_part_A_B, representing the full score across both forms; (2) Total_score_1_5_A, reflecting responses to key functional items in Form A; and (3) Total_score_1_2_4_6_7_9_B, corresponding to behavioral and sensory subscale items in Form B. Each of these scoring subsets was used to capture different aspects of the child’s feeding behavior. The internal consistency and test–retest reliability of the measurements were examined, and previous studies have demonstrated acceptable reliability levels for these subscales, supporting their use in clinical and research contexts.

Correlations between the Child Eating Behaviour Questionnaire (CEBQ) and PASS-FP were also considered for criterion validity, as were comparisons between tube-fed, partially tube-fed, and exclusively oral-fed children. The results of this study showed that the T-PASS-FP had significant positive correlations with the EF (Desire for Food) and FF (Food Fussiness) subscales of the CEBQ Π = 0.002; Π = 0.012, respectively). The positive correlation between the T-PASSF subscale was marginally significant (Ι = 0.053) [3].

### 2.4. Statistical Analysis

Kolmogorov–Smirnov and Shapiro–Wilk tests were used to screen variables before primary operations for data input accuracy, missing values, probable outliers, and distribution (skewness and kurtosis). All normally distributed variables were expressed as means (Ms) and standard deviations (SDs). Similarly, variables that did not follow a normal distribution were described using the median and interquartile range. To compare the means of responses for the PASS-FP between the two major groups, an independent sample t-test was applied. Furthermore, a one-way ANOVA test was used to assess mean differences across the study’s subgroups. Thus, we measured the study’s primary group effects between the typical feeding group, the oral-fed group, the partially oral-fed with supplementation, and the tube-fed subgroups and followed their medical diagnosis (syndromes, acquired disorders, developmental disorders, and cerebral palsy groups).

The validity of the Greek version of the PASS-FP was evaluated using the Content Validity Index (CVI). This involved five speech–language pathologists (SLPs) who specialize in pediatric dysphagia. For each item, the Item-CVI (I-CVI) was calculated by dividing the number of SLPs in agreement by five, the total number of experts. The overall Scale-CVI (S-CVI) was determined by summing the I-CVIs and dividing the value by 10. Research guidelines state that outstanding content validity is indicated by I-CVIs of 0.78 or higher and S-CVIs of 0.8 or above [37,38].

Furthermore, the reliability analysis of the PASS-FP in Greek was estimated using (a) Cronbach’s alpha coefficient for the internal consistency of its dimensions and (b) Spearman’s ρ correlation coefficient for its test–retest reliability. Moreover, to determine the cut-off scores of the PASS-FP- Gr questionnaire, a Receiver Operating Characteristic (ROC) analysis was computed for the mean score of answers between parents of children without feeding and swallowing disorders and parents of children with feeding and swallowing disorders.

All reported *p*-values are two-tailed, with a statistical significance threshold set at 0.05. The statistical analyses were performed using IBM SPSS Statistics version 28 (IBM Corp., 2021, Armonk, NY, USA) and RStudio version 2022.02.3+492 “Prairie Trillium” (RStudio Team, 2020, Boston, MA, USA, URL http://www.rstudio.com/) (accessed on 2 March 2022) [39].

## 3. Results

### 3.1. Demographic Data

This study included 142 children with different neurological and medical diagnoses and were separated into three subgroups: the oral-fed, the partially oral-fed with supplementation, and the tube-fed.

### 3.2. The Group Effect for the PASS-FP-Gr Questionnaire

Kruskal–Walli’s test found significant variations in median values between the three groups (the oral-fed, the partially oral-fed with supplementation, and the tube-fed). The statistical significance of the differences detected in the median scores across the three groups is indicated by a low *p*-value, (*p* < 0.001), which is far lower than the conventional significance level of 0.05. Comparing the oral-fed group (52.0) to the partially oral-fed with supplementation group (43.5), the difference in median score is notable, with the former scoring 8.5 points higher. The median score of the partially oral-fed with supplementation group (43.5) is 16.5 points higher than that of the tube-fed group (27.0) (as presented in Table 1). A series of Mann–Whitney tests revealed substantial differences between each group and the others. More specifically, there was a significant difference in median values (*p* < 0.001) between the oral and partially fed groups. Similarly, there were significant differences (*p* < 0.001) in the median values between the partially fed and tube-fed groups and between the oral- and tube-fed groups. These findings demonstrate how the measured variable varied significantly between the three groups.

**Figure 2:**
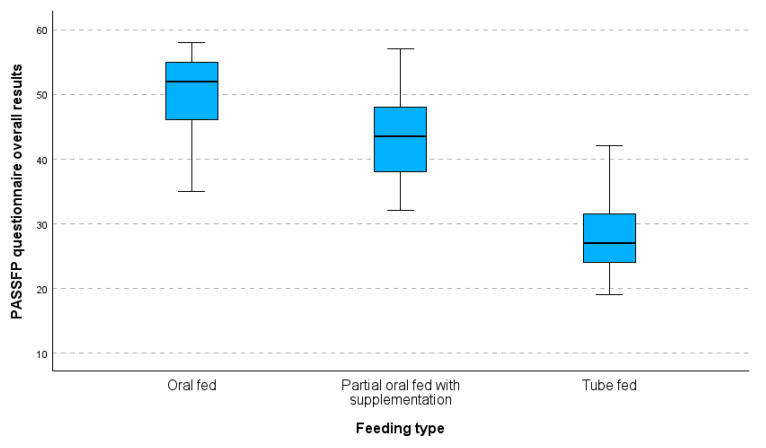
Boxplot of PASS-FP-Gr questionnaire overall results by feeding type.

Figure 2 shows a boxplot of the overall results from the PASS-FP-Gr questionnaire categorized by feeding type. The median scores for each feeding type are indicated, providing an insight into the central tendency of the responses. The boxplot indicates a clear pattern in the median values of the three feeding groups: the oral-fed group has the highest median value, followed by the partially oral-fed group with supplements, and finally, the tube-fed group, which has the lowest median value. As we move from the oral-fed group to the tube-fed group, the variability in values increases. The tube-fed group exhibits both the widest range of values and the highest number of deviations.

Thus, there were noticeable variations when IQRs and median scores were compared between the three feeding types. Not only did the tube-fed children have a lower median score, but they also showed more variance, which showed that different ways of feeding had different effects on their PASS-FP scores.

The one-way Anova method was conducted to compare the sub-scores of the PASS-FP-Gr questionnaire between the study’s subgroups that were split by the diagnosis of the feeding process. The results of the ANOVA analysis indicate significant differences in mean values between the three feeding groups. For the total score for parts A and B, F (2,139) = 76.509, *p* < 0.001, demonstrating that the feeding process significantly influences these values. Further evidence that the feeding process has a considerable impact on these values comes from F (2,139) = 134.756, *p* < 0.001, for total_score_1__5_A. Finally, total_score_1_2_4_6_7_9_B also shows significant differences between groups, with an F (2,139) = 27.769, *p* < 0.001, representing that the feeding process significantly impacts these scores. The total PASS-FP-Gr score was used as the dependent variable in the univariate analysis of variance, whereas the group (oral-fed, partially oral-fed with supplementation, and tube-fed) served as the independent variable. All the results are summarized in Table 2.

### 3.3. Internal Consistency and Reliability Measures for the PASS-FP-Gr Questionnaire

The PASS-FP-Gr questionnaire was adapted for this study, and some items were excluded from analysis for specific subgroups. To assess the validity of the remaining 13 questions, an analysis was performed that removed the scores for items 1 and 2 related to oral feeding from the total score for the children who were either partially or exclusively tube-fed. This adjustment is clearly reflected in the table legends.

To examine the construct validity of items 1 and 2, Spearman’s ρ correlations were calculated between these two items and the revised total score (excluding the two items). The results were statistically significant for children who were either partially or exclusively tube-fed. For item 1 (Spearman’s ρ = 0.292, *p* = 0.010) and item 2 (Spearman’s ρ = 0.282, *p* = 0.013), both correlations were statistically significant, indicating a moderate association between each item and the overall feeding performance score in the tube-fed and partially tube-fed groups.

Internal consistency was measured using Cronbach’s alpha. The alpha coefficient for all three groups (*n* = 142) was 0.766, indicating acceptable internal consistency. For the two groups who were either partially or exclusively tube-fed children (*n* = 77), it was 0.706, which also falls within an acceptable range (Table 3).

Test–retest reliability was also high. For the 142 children who were administered the second questionnaire, the mean score of the first administration was 44.9, SD 8.9, and the mean score of the second administration was 44.9, SD 8.9. Due to the identical means and standard deviations, a paired-sample t-test could not be conducted. However, a perfect Spearman’s correlation (ρ = 1.0) was observed between the two administrations, indicating excellent test–retest reliability. No *p*-value was calculated due to the lack of variability.

#### 3.3.1. CVI Analysis

To establish the content validity of the PASS-FP in the Greek language, the S-CVI was computed and was equal to 1. The clearance of the questionnaire and the CVI for all items was 1, and the total agreement was 29.

#### 3.3.2. ROC Analysis for the PASS-FP-Gr Questionnaire

**Figure 3:**
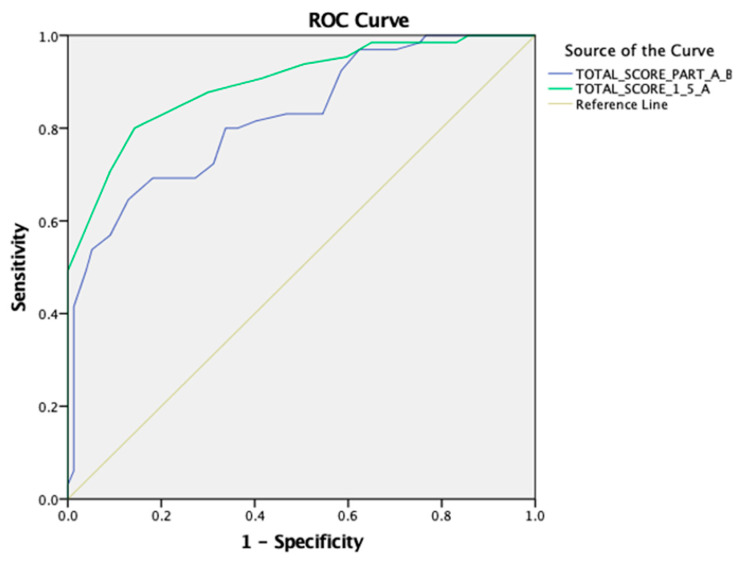
An ROC curve for the PASS-FP-Gr questionnaire for the total score of parts A and B and the total score of part A.

The cut-off thresholds for the PASS-FP-Gr total score of parts A and B, part A, and the concept validity were determined using an ROC analysis. A statistically significant positive discrimination of the total score of parts A and B was revealed [AUC 0.824 (95% CI: 0.756–0.892), *p* < 0.001]. The cut-off point was equal to 49.00 with a sensitivity of 0.646 and a 1-specificity of 0.13. Additionally, a statistically significant positive discrimination between the total score of part A revealed [AUC 0.897 (95% CI: 0.845–0.948), *p* < 0.001]. The cut-off point was equal to 18.00 with a sensitivity of 0.800 and a 1-specificity of 0.143 (as shown in Figure 3).

## 4. Discussion

The present study offers preliminary but crucial evidence that the Greek version of the PASS-FP is a valuable scale for assessing severe feeding problems in children. Good psychometric qualities, such as excellent internal consistency, test–retest reliability, and discrimination among feeding groups, guaranteed reliability in the clinical context.

### 4.1. Complex Nature of Feeding Difficulties

The comprehensive characteristics of the PASS-FP, including behavioral, psychological, nutritional, and medical aspects, denote best practices that advocate for multidisciplinary approaches in managing pediatric feeding disorders. Speech–language pathologists’ contribution to validating the Greek version emphasized its clinical relevance and applicability to the existing literature [33,40].

This study fills an important gap by providing a validated assessment tool for children with feeding and swallowing difficulties in the Greek Cypriot population. Standardized questionnaires for the pediatric population demonstrating feeding difficulties necessitating certain feeding and swallowing procedures were not publicly accessible while this research was conducted [41,42]. The Greek version of the PASS-FP offers a comprehensive context of assessment, while it gathers detailed insights from the parents regarding their child’s feeding behaviors across various contexts.

These different approaches to feeding are clearly distinguished by the PASS-FP-Gr, which is evident from the trend of scores among the oral-fed, partially oral-fed with supplementation, and tube-fed groups. The higher and more homogeneous scores in the oral-fed group, contrasted against the variability in the partially oral-fed and tube-fed groups, reflect the sensitivity and discriminant power of the scale.

### 4.2. Psychometric Properties

Cronbach’s alpha value for the PASS-FP-Gr revealed good internal consistency: 0.766. Test–retest reliability was excellent. These findings indicated that the scale items consistently measured the intended construct and provided stable results over time, an important factor in clinical practice where repeated assessments are performed to monitor changes and the effectiveness of the intervention. The reported reliability of the PASS-FP -Gr agrees with other validation studies of the PASS-FP in languages other than English, thus supporting the robustness of the instrument across diverse cultural contexts [3]. While these results are promising, it is important to consider the limitations related to the number of participants within each feeding subgroup. In particular, the tube-fed group had a smaller sample size, which may have influenced the stability of the internal consistency and validity findings for that subgroup. Although the overall psychometric properties of the scale were strong, future studies with larger and more balanced subgroup samples are needed to confirm and expand on these results.

Additionally, the boxplot depicts the major differences in PASS-FP-Gr scores for the three feeding subgroups whereby the oral-fed group has the highest and least variable values, the partially oral-fed with supplementation is more scattered, and the tube-fed has the lowest and most scattered. The results of the boxplots verify the Kruskal–Wallis’s test in reporting the presence of statistically significant differences between these groups

### 4.3. Discriminatory Ability

Through the ROC analysis, the total score of parts A and B revealed an AUC of 0.824 and obtained a *p*-value < 0.001, indicating excellent discriminatory power. This is an important finding since the results showed that the scale can differentiate between those with different feeding difficulties. The early classification of children with feeding difficulties is necessary for early intervention to prevent feeding problems from negatively affecting a child’s nutritional status, growth, and general development. With this high discriminant ability, the PASS-FP-Gr could be a strong and trustworthy tool in clinical practice, helping healthcare professionals make sound decisions about the need and form of interventions.

### 4.4. Comparison with Previous Studies

The findings of this study are consistent with the Turkish version of the PASS-FP, validated by Yazici-Gulay et al., 2021, which had very good psychometric properties and proved to be an effective screening instrument for children’s feeding problems and the validation study by Crist et al., 2004 [3,18]. Such consistency across different cultural adaptations underlines the strength and psychometric properties of the PASS-FP as a tool in assessing feeding problems among various populations. It also brings out the need for cultural adaptation to ensure the questionnaire’s relevance and accuracy in varying cultural contexts.

### 4.5. Criterion Validity

Both researchers used different external approaches to evaluate the criterion validity of feeding approaches. For the Greek PASS-FP, speech–language pathologists evaluated the Greek PASS-FP’s content validity index and determined that it was sufficiently significant to suggest strong content validity. The criterion validity in the Turkish study used the Children’s CEBQ, and its subscales of Food Responsiveness, Enjoyment of Food, and Food Fussiness were positively correlated at *p* < 0.05 [3,43]. This validation approach aligns with the methodology employed in the Greek study to ensure an accurate assessment of the questionnaire’s validity.

### 4.6. Cultural Adaptation and Relevance

To maintain the scale’s relevance and integrity, a thorough and systematic process was undertaken for the translation and cultural adaptation of the PASS-FP for both Greek and Turkish contexts [3]. In both studies, there were forward and backward translations, coupled with reviews and adjustments to the translated versions agreed upon by multidisciplinary teams. Given that the PASS-FP is universally applicable for evaluating severe eating difficulties in children, it is evident that the results have been similar throughout various studies and that the tool is effective in a variety of cultural contexts.

### 4.7. Strengths and Limitations

The Greek translation of the PASS-FP demonstrates strong validity as a comprehensive instrument for accurately capturing the medical, nutritional, psychological, and behavioral dimensions of feeding difficulties. It has high internal consistency and is highly test–retest reliable. This underlines the psychometric properties of the measure of feeding problems in children. Brought out by the tool’s discriminant ability, it separated, without any overlap, oral-fed, partially oral-fed with supplementation, and tube-fed children, thus showing sensitivity and specificity. The cultural adaptation in the Greek-Cypriot setting makes the tool relevant and accurate in reflecting specific feeding difficulties and swallowing weaknesses unique to this population. It is also important to have SLPs and other experts involved in the validation process, emphasizing the need for a multidisciplinary approach which is essential when creating and implementing any assessment tools for feeding and swallowing processes. More importantly, Greek PASS-FP bridges a serious gap in standardized assessment tools for the Greek-Cypriot population and becomes a valuable tool for both the clinician and the researcher.

The limitations include sample size and diversity, as the findings are based on a relatively small and specific population of participants; further research with larger and more diverse groups is needed to generalize these findings. Moreover, since the sample was specifically chosen from clinical and educational settings, there is a chance that it does not fully reflect the broader population of Greek-speaking children with feeding difficulties. This may introduce some selection bias and limit how widely the findings can be applied. This study’s design is cross-sectional, and therefore, this limits the conclusion to be drawn on the longitudinal effectiveness of interventions based on PASS-FP assessments. Long-term studies are also necessary to assess how the tool affects treatment results over an extended period. Importantly, some of the feeding subgroups—especially the tube-fed group—had a relatively small number of participants. This may have affected how stable and reliable the results were for those specific groups. Larger subgroup samples are needed to confirm these psychometric findings. Additionally, a potential bias arises from the parent and caregiver reports in assessing feeding behaviors, since such reports are subjective. This would be further complemented by direct clinical observation and objective measures to enhance the psychometric property of the assessments. Finally, while content validation involved five experienced speech–language pathologists specializing in pediatric dysphagia, increasing the number of experts to at least seven—as recommended in methodological guidelines—could improve the reliability of content validity indices. This should be considered in future validation studies.

### 4.8. Future Directions

The PASS-FP, translated into Greek, was established as a valid, reliable, and comprehensive instrument for assessing severe feeding and swallowing problems in Greek Cypriot children. Its integration in clinical practice may drive significant improvements in the quality of care and outcomes for children with feeding difficulties. Future studies should adopt prospective designs to monitor the progress of interventions informed by PASS-FP assessments and to examine its applicability in other Greek-speaking populations. The development and use of standardized assessment tools remain essential for advancing the management of pediatric feeding and swallowing disorders, and the PASS-FP represents a valuable contribution in this regard.

## 5. Conclusions

The Greek version of the PASS-FP has demonstrated strong content validity and reliability as a tool for identifying severe feeding and swallowing problems in children within the Greek Cypriot population. Its good internal consistency and test–retest reliability confer high discriminatory power and dependability, establishing it as a valuable resource for both researchers and clinicians. By facilitating early identification and targeted intervention, this instrument holds promise for improving outcomes in children with feeding and swallowing difficulties.

This study represents an important first step in addressing the critical need for validated, culturally appropriate assessment tools in pediatric feeding and swallowing evaluation. Future research should seek to validate this instrument in larger, more representative samples and include additional forms of validation, such as factor analysis to enhance its generalizability and applicability across diverse clinical settings. The successful adaptation into Greek sets a precedent for further translations and cultural adaptations, contributing to the global advancement of knowledge in pediatric feeding difficulties.

## Figures and Tables

**Table 1:** The PASS-FP-Gr questionnaire total score for the study’s subgroups (parts A and B).

Type of Feeding Process	*n*	Mean	Median	SD	Min	Max
Oral-fed	65	50.1	52.0	5.9	35.0	58.0
Partially oral-fed with supplementation	62	43.4	43.5	6.2	32.0	57.0
Totally tube-fed	15	28.7	27.0	7.2	19.0	42.0
Total	142	44.9	46.5	8.9	19.0	58.0

Footnote: The total score of the PASS-FP as the dependent variable and group (totally tube-fed, partially tube-fed, totally orally fed); SD = standard deviation; Min = minimum score; Max = maximum score.

**Table 2:** Group Effect of the PASS-FP-Gr Questionnaire Total sub-score between the study’s Subgroups.

	Oral-Fed (*n* = 65)	Partially Oral-Fed with Supplementation (*n* = 62)	Tube-Fed (*n* = 15)	F (2,139)	*p*
	M (SD)	M (SD)	M (SD)		
Total_score_part_A_B	50.09 (5.88)	43.40 (6.19)	28.73 (7.15)	76.509	<0.001
Total_score_1_5_A	20.20 (2.51)	16.17 (2.79)	8.06 (2.65)	134.756	<0.001
Total_score_1_2_4_6_7_9_B	20.78 (3.90)	17.56 (4.19)	12.20 (5.50)	27.769	<0.001

Footnote: M = mean; SD = standard deviation.

**Table 3:** Internal consistency and item—total correlations for PASS-FP-Gr questionnaire.

Group	Cronbach’s α	Spearman’s ρ (Item 1)	*p*-Value	Spearman’s ρ (Item 2)	*p*-Value
Full sample (*n* = 142)	0.766	-	-	-	-
Tube-fed and partially oral-fed (*n* = 77)	0.706	0.292	0.010	0.282	0.013

Footnote: Items 1 and 2 were excluded from total score calculations for tube-fed and partially orally fed groups due to non-applicability. Spearman’s ρ values reflect correlations between each excluded item and the revised total score (13 items). Cronbach’s α indicates internal consistency for each group.

## Data Availability

The data underlying this article cannot be shared publicly because of the privacy and confidentiality of the study participants, which are protected. The data of this study are available upon reasonable request from the corresponding author and the signing of a data transfer agreement.

## Abbreviations

The following abbreviations are used throughout this manuscript:

ASD	Autism Spectrum Disorder
CEBQ	Child Eating Behavior Questionnaire
CVI	Content Validity Index
GT	Gastrostomy Tube
M	Mean
PASSFP	Pediatric Assessment Scale for Severe Feeding Problems
ROC	Receiver Operating Curve
SD	Standard Deviation
SLP	Speech–Language Pathologist
SPSS	Statistical Package for the Social Sciences
WHO	World Health Organization
